# Lactic Acid Bacteria-Fermented Natural Products: Mechanisms and Evidence for Ageing- and Stem-Cell-Related Outcomes

**DOI:** 10.3390/foods15183252

**Published:** 2026-09-15

**Authors:** Luwei Wang, Sa-ouk Kang, Rui Liu, Bo Sun

**Affiliations:** 1 School of Food Engineering, Yantai Engineering Research Center of Food Green Processing and Quality Control, Ludong University, Yantai 264025, China; 2 Laboratory of Biophysics, School of Biological Sciences, and Institute of Microbiology, Seoul National University, Seoul 151-742, Republic of Korea; kangsaou@snu.ac.kr; 3 State Key Laboratory of Digital Medical Engineering, Southeast University, Nanjing 210096, China

**Keywords:** lactic acid bacteria, fermentation, anti-ageing, stem cell health, bioactive metabolites

## Abstract

Ageing is driven by chronic inflammation, oxidative stress, and stem cell exhaustion, posing a major global health challenge. Lactic acid bacteria (LAB) fermentation is being investigated as a potential biotechnology to enhance the bioavailability and functionality of natural products, especially polyphenols and bioactive phytochemicals with anti-ageing potential. This review synthesizes recent advances in LAB-fermented natural products and their roles in modulating longevity pathways and stem cell regeneration. LAB fermentation can alter complex plant matrices by releasing or biotransforming substrate-bound phytochemicals and by generating metabolites such as organic acids, peptides, and, in some systems, short-chain fatty acids (SCFAs), cyclic dipeptides (CDPs), or bacterial extracellular vesicles (BEVs); the contribution of each component requires case-specific verification. In cellular and animal models, candidate mechanisms include modulation of nuclear factor kappa B (NF-κB), nuclear factor erythroid 2-related factor 2 (Nrf2), and AMP-activated protein kinase–sirtuin 1 (AMPK–SIRT1) signaling, although causal active components are often unresolved. Some LAB-fermented preparations or LAB-derived components have been associated with stem-cell-related outcomes in skin, bone, and neural cell or animal models. CDPs and BEVs are candidate fermentation-associated mediators, but evidence for their independent effects on human ageing remains preliminary. Collectively, LAB-fermented natural products are a promising research area for functional foods and nutraceuticals, but their efficacy and safety for ageing-related outcomes require clinical validation. Future research should focus on standardizing fermentation conditions, identifying active metabolites, and validating their clinical efficacy in human studies.

## 1. Introduction

Ageing is a multifactorial biological process characterized by progressive functional decline and increased risk of chronic diseases. A central challenge in geroscience is identifying interventions that slow ageing by targeting its key mechanisms, such as oxidative stress, chronic inflammation, and stem cell exhaustion [1,2,3]. Traditional Chinese medicine (TCM) and other botanical medicines have long been used for vitality and longevity, but many natural active compounds in their crude form have limited bioavailability or potency [4]. Recent advances have highlighted lactic acid bacteria (LAB) fermentation as a promising strategy to enhance the bioactivity of these natural compounds. By fermenting herbs or plant materials with probiotic LAB strains, researchers can boost the content of bioactive metabolites, transform precursors into more absorbable forms, and even generate novel anti-ageing molecules [5,6,7]. Additional fermentation-associated mechanisms have been reported [8]. For example, fermentation of the herb Lespedeza cuneata with *Lactiplantibacillus pentosus* increased its flavonoid content about 2.5-fold and significantly improved antioxidant and anti-ageing activity [9]. Similarly, Fermentation of apple pomace (AP) with *Lactiplantibacillus plantarum* KKP 1527 significantly enhances total phenolic content (by approximately 30%), antioxidant activity, and enriches polyphenols such as gallic acid, protocatechuic acid, and procyanidin B2, highlighting its potential as a low-cost natural antioxidant source with broad industrial applications [10]. These examples illustrate that LAB fermentation can modify the chemical composition and potential bioaccessibility of natural products; efficacy must be assessed for each strain–substrate–product combination. In this review, we survey recent progress in LAB-fermented compounds for anti-ageing benefits, with a focus on mechanistic insights and impacts on stem cell function. We also examine two special categories of LAB-derived metabolites—cyclic dipeptides (CDPs) and bacterial extracellular vesicles (BEVs)—and discuss their roles in modulating ageing and stem cell biology. Finally, we consider the challenges and future directions for translating these findings into interventions that promote healthy ageing.

Scope and literature selection. This narrative review was informed by English-language reports identified in PubMed, Web of Science, and Scopus from January 2013 to June 2025, using combinations of ‘lactic acid bacteria’, ‘fermentation’, ‘ageing/aging’, ‘stem cell’, ‘cyclic dipeptide/diketopiperazine’, and ‘extracellular vesicle’. We prioritised studies that identified the fermentation system and reported a biological endpoint; evidence is interpreted here according to whether it arose from human, animal, cellular, or mechanistic work.

Selection boundaries and limitations. The review included original human, animal, cellular, and chemical or mechanistic reports only when the LAB strain or fermentation system and a relevant endpoint could be identified; key reviews and consensus statements were used to contextualise terminology or broader concepts. Reports without sufficient information to identify the intervention or endpoint were not used as evidence for a biological claim. This was not a systematic review or meta-analysis because the products, exposures, and outcomes were too heterogeneous for pooled inference; consequently, selection bias cannot be fully excluded.

Terminology. In this review, ‘probiotic’ is reserved for live microorganisms with a demonstrated health benefit, whereas a LAB-fermented product is the food or botanical matrix produced by fermentation. CDPs and other purified fermentation-derived metabolites are not themselves postbiotics under the ISAPP definition; a postbiotic is a preparation of inanimate microorganisms and/or their components that confers a health benefit on the host [11]. BEVs are therefore discussed as a distinct bacterial component whose status depends on the preparation and the evidence for host benefit.

## 2. Lactic Acid Bacteria Fermentation of Natural Compounds

LAB are widely used in food fermentations and as health-promoting probiotics. During fermentation, LAB enzymes biotransform complex substrates in plant materials into smaller, more bioactive compounds [12,13]. This process can modify the chemical profile and, in some cases, the bioaccessibility of herbal preparations. LAB fermentation can increase measured free or extractable phenolics and peptides; such changes commonly reflect release from bound forms, hydrolysis, or biotransformation rather than de novo production of plant polyphenols. Fermentation of kiwifruit pulp with *Lactiplantibacillus plantarum* significantly enhanced antioxidant activity and increased the levels of phenolics and flavonoids, particularly protocatechuic acid, while reshaping the phenolic profile and metabolite composition, underscoring its potential for health-promoting applications [14]. Likewise, probiotic fermentation of ginseng is known to convert ginsenoside glycosides into rare metabolites (e.g., compound K, Rh1) with higher bioavailability and bioactivity [15]. In a human pharmacokinetic study, fermented ginseng increased plasma compound K exposure relative to unfermented ginseng; this supports altered absorption, but does not establish an anti-ageing clinical benefit [15]. LAB also secrete hydrolytic enzymes (proteases, glycosidases) that break down macromolecules [5]. This can release bound antioxidants or create new metabolites. For instance, fermentation of soy by LAB liberates aglycone isoflavones and generates small peptides [16]. *Lactiplantibacillus plantarum HFY09*-fermented soymilk showed elevated free isoflavones and peptides and was found to delay ageing in mice via antioxidative mechanisms [17]. In general, LAB fermentation increases the yield of easily absorbed, biologically potent compounds while reducing toxic or inert ingredients. It can also produce short-chain fatty acids (SCFAs), exopolysaccharides, and organic acids that benefit the host [18,19]. For example, fermenting medicinal fungus Cordyceps with Pediococcus increased levels of SCFAs and cordycepin, resulting in heightened immunomodulatory activity [20]. Whether fermentation improves a biological outcome relative to the unfermented substrate remains formulation-specific and should be established using matched controls. Most available anti-ageing evidence remains preclinical, with heterogeneous endpoints and limited information on dose–response relationships or long-term exposure.

**Figure 1:**
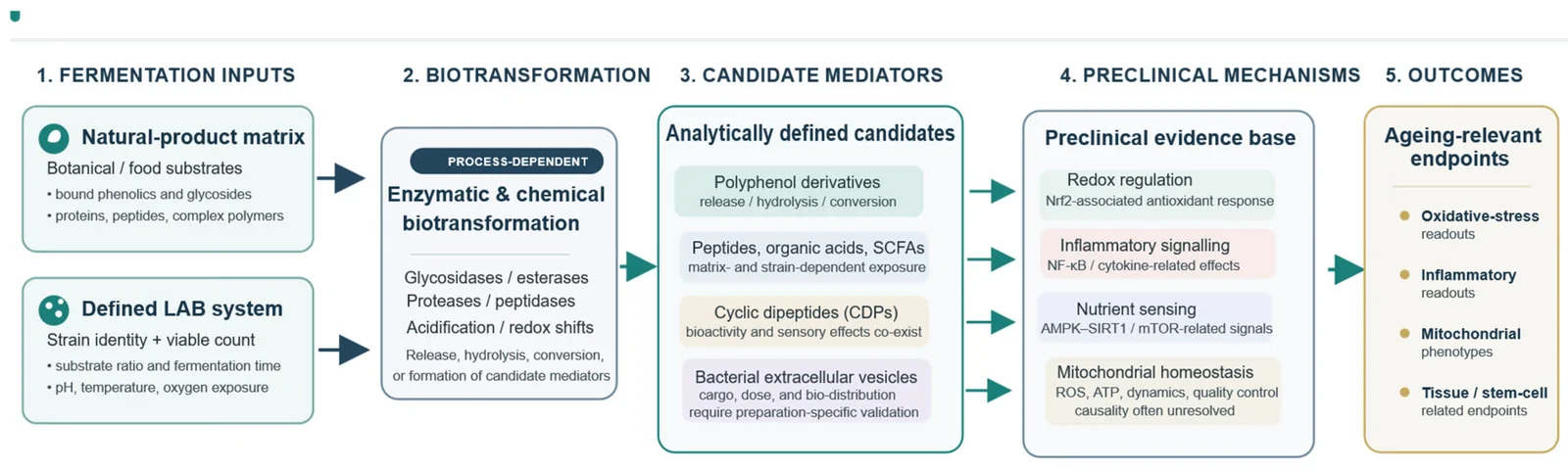
Conceptual framework linking lactic acid bacteria (LAB) fermentation of natural products to candidate ageing-related cellular effects. Botanical or food-derived matrices contain bound phenolics, glycosides, proteins, and other complex dietary polymers. Fermentation with a defined LAB strain under controlled process conditions can remodel this matrix through enzymatic hydrolysis, acidification, and metabolic conversion. The resulting preparation may contain altered polyphenol derivatives, peptides, organic acids and short-chain fatty acids (SCFAs), cyclic dipeptides (CDPs), and/or bacterial extracellular vesicles (BEVs); the identity and abundance of these candidate mediators are product-specific. In cellular and animal studies, selected preparations or components have been associated with redox regulation, modulation of inflammatory signalling, mitochondrial homeostasis, and stem-cell-related pathways involving Nrf2, NF-κB, AMPK–SIRT1/mTOR, and related networks. These changes are assessed using oxidative/inflammatory, mitochondrial, and tissue- or stem-cell-related readouts. Arrows indicate reported or hypothesised relationships rather than universal causal effects. The dashed evidence boundary highlights that most available data are preclinical. Translation to health claims requires rigorous product standardisation, chemical and biological characterisation, exposure and long-term safety assessment, and controlled human studies with prespecified clinical efficacy and safety outcomes.

An overview of the proposed links between defined LAB fermentation, candidate fermentation-associated mediators, ageing-relevant cellular processes, and the translational evidence boundary is presented in Figure 1.

## 3. Anti-Ageing Mechanisms of LAB-Fermented Products

Oxidative stress is a frequently investigated pathway through which LAB-fermented preparations may influence ageing-related phenotypes in preclinical models. Oxidative damage accumulates with age and drives cellular senescence. LAB-associated metabolites have shown antioxidant activity in experimental systems, although their bioavailability and in vivo contribution depend on the formulation and host context [21]. In the D-galactose ageing mouse model, supplementation with *Lactiplantibacillus plantarum*-fermented soymilk significantly increased antioxidant enzyme activities (superoxide dismutase, catalase, glutathione peroxidase) in multiple organs, while decreasing markers of oxidative damage such as malondialdehyde and advanced glycation end-products [17]. Molecular analyses revealed that the fermented soymilk activated the Nrf2/KEAP1 pathway, a master regulator of cellular antioxidative responses. Nrf2 and its downstream targets (HO-1, NQO1, GCLM, SOD2, etc.) were upregulated in liver and spleen of treated mice, indicating enhanced antioxidant capacity. Consistently, probiotic LAB can directly trigger Nrf2 pathways in host tissues: for example, oral *Levilactobacillus brevis* elevated Nrf2 and HO-1 expression in the gut, leading to reduced inflammatory injury in an oxidative stress model [22]. These observations support a hypothesis of redox modulation, but do not yet establish a durable anti-ageing effect in humans.

Suppression of chronic inflammation is another key mechanism. Ageing is often accompanied by “inflammageing,” a low-grade pro-inflammatory state deleterious to tissues. Some LAB-associated metabolites have modulated immune signalling in experimental systems, but the direction and magnitude of the response depend on the strain, matrix, dose, and model. For instance, certain cyclic dipeptides interfere with the NF-κB pathway, thereby reducing the production of pro-inflammatory cytokines [23]. Cyclo(L-Phe-L-Pro), a cyclic dipeptide reported in several microbial systems, was shown to inhibit NF-κB activation in macrophages and downregulate inflammatory mediators like TNF-α and IL-6 [24]. In vivo, probiotic interventions have mirrored these effects. Mice receiving LAB-fermented foods often exhibit lower levels of IL-1β, TNF-α and other cytokines associated with chronic inflammation, along with higher levels of anti-inflammatory cytokines (e.g., IL-10, TGF-β) or regulatory T-cell responses [25]. The available studies vary substantially in the strain, food matrix, model, and inflammatory endpoint; therefore, they do not yet support a common anti-inflammatory effect for LAB-fermented products. Accordingly, anti-inflammatory effects should be presented as context-dependent preclinical observations rather than established healthy-ageing outcomes.

Modulation of nutrient-sensing pathways such as mTOR, AMP-activated protein kinase (AMPK), and sirtuins [26] also underlies the anti-ageing action of LAB-fermented compounds. These pathways regulate metabolism, protein synthesis, and autophagy, all of which impact longevity. LAB are known to produce various amino acids, polyamines, and other metabolites that can influence these signaling cascades [27]. While direct evidence of LAB fermentation inhibiting mTOR in mammals is still emerging, there are intriguing parallels: dietary restriction and pharmacological mTOR inhibitors are known to extend lifespan by boosting autophagy and reducing inflammation [28]. LAB fermentation products may achieve a similar end via AMPK activation. SCFAs like butyrate (often increased in fermented herbal mixtures) activate AMPK, which in turn suppresses mTOR and promotes cellular cleanup processes [29,30,31]. Additionally, probiotics can increase levels of NAD^+^ or polyphenols that activate SIRT1, a longevity-associated deacetylase [32]. In an Alzheimer’s mouse model, a multi-strain LAB formulation (SLAB51) markedly reduced oxidative brain damage by activating SIRT1-dependent pathways [33]. SIRT1 activation enhances mitochondrial biogenesis and stress resistance in neurons, consistent with improved cognitive function [34]. Together, these findings identify nutrient-sensing pathways as plausible mechanistic targets, but the available studies do not demonstrate that LAB-fermented products reproduce the effects of caloric restriction or slow ageing in humans.

Mitochondrial protection and metabolic enhancement are further mechanisms linked to LAB-fermented interventions. Mitochondrial dysfunction is a hallmark of ageing, leading to energy deficits and more ROS production [35,36]. Some fermented preparations or LAB-derived components have improved mitochondrial readouts in cellular or animal experiments. For example, treatment of oxidatively stressed cells with LAB-derived EVs increased mitochondrial fusion and ATP production, which protected the cells from stress-induced senescence [37]. Likewise, aged animals given certain probiotic strains show improved mitochondrial enzyme activities in tissues and higher ATP levels, consistent with altered cellular energy metabolism [38]. LAB can also produce B vitamins and amino acids that fuel mitochondrial function. Furthermore, some fermented herbal extracts stimulate FOXO and other transcription factors that upregulate mitochondrial quality control and antioxidative enzymes [39]. These findings warrant mechanistic follow-up, but do not establish prevention of mitochondrial ageing in humans.

**Figure 2:**
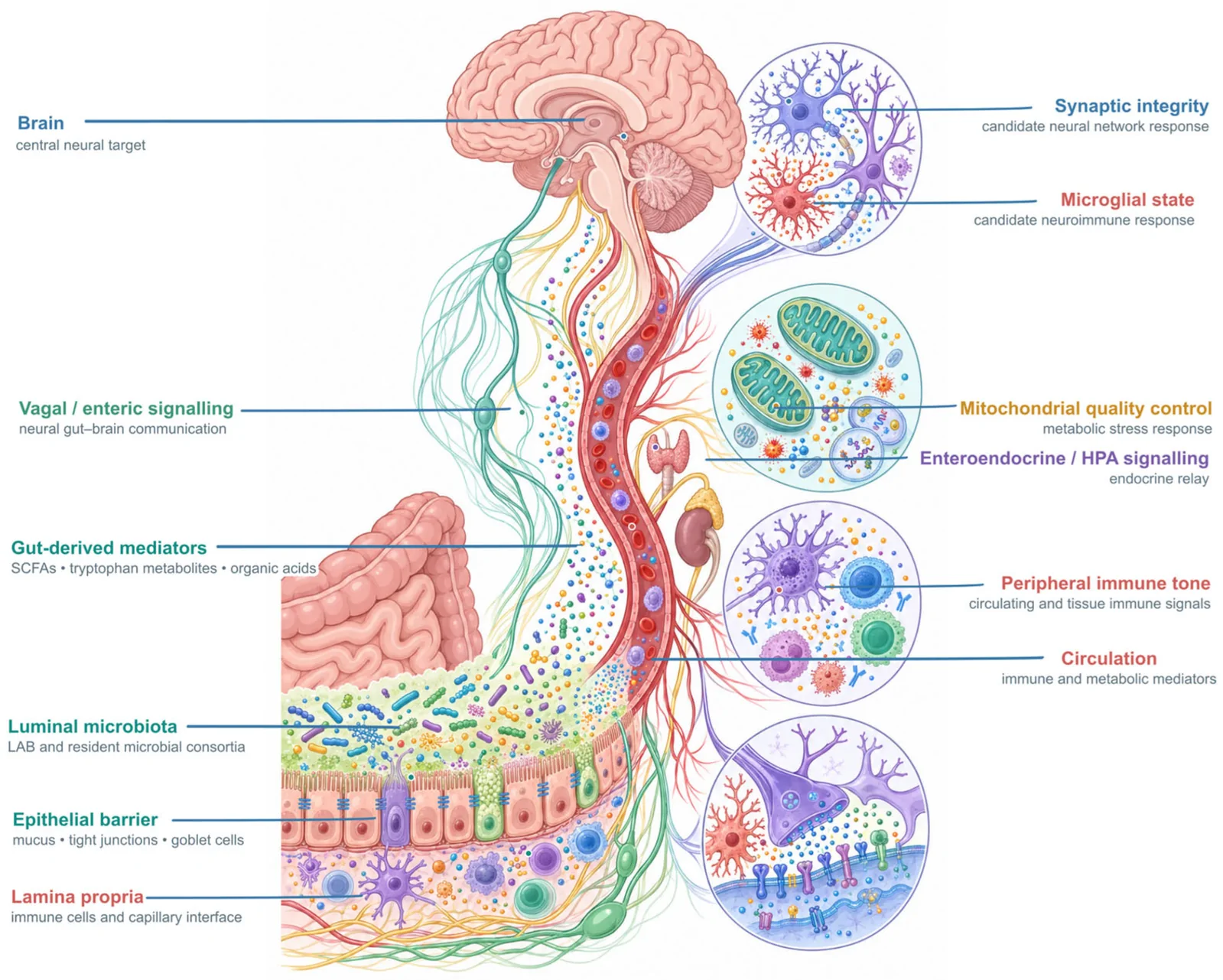
Conceptual mechanism map of gut–brain-axis routes potentially linking LAB-fermented products to ageing-related phenotypes. The intestinal barrier and resident microbiota (lower left) provide microbial and host-derived signals—including short-chain fatty acids, tryptophan-derived metabolites and organic acids—that may be relayed through enteric/vagal connections, circulating immune and metabolic mediators, and endocrine pathways to the brain. The peripheral cellular vignettes denote candidate downstream processes—microglial and immune tone, mitochondrial quality control and synaptic integrity—reported mainly in experimental settings. This is a hypothesis-generating framework, not a universal causal model. Clinical findings discussed below are product- and outcome-specific and do not establish general healthy-ageing efficacy.

An additional integrative route through which fermentation-associated signals may influence ageing-related phenotypes is the gut–brain axis (Figure 2). The conceptual map links the microbe-rich intestinal barrier to neural, vascular, immune and endocrine relays, and then to candidate cellular responses in the brain and peripheral tissues. Strain- and matrix-dependent changes in the intestinal milieu can alter the availability of short-chain fatty acids, tryptophan-derived metabolites, organic acids and other microbial products, while also affecting epithelial-barrier and immune tone. In experimental settings, downstream correlates include altered microglial activation, neurotrophic signalling, mitochondrial stress responses and cognitive function. In naturally aged mice, *Lactobacillus plantarum* LLY-606 improved cognitive outcomes together with barrier measures, lower inflammatory cytokines and predicted enrichment of tryptophan-, propanoate- and synapse-related pathways [40]. This supports a product-specific mechanistic model, not a general anti-ageing effect.

Human intervention studies provide early but mixed translational signals. In older adults with declining memory, a randomized placebo-controlled trial reported improvements in selected memory endpoints after 12 weeks of *Lactiplantibacillus plantarum* OLL2712 [41]. In a 4-week randomized trial of 66 adults over 70 years with low-grade inflammation, *L. plantarum* HEAL9 lowered faecal calprotectin but did not significantly reduce serum C-reactive protein or improve cognitive outcomes [42]. Broader gut-directed interventions provide complementary context: a multicentre, double-blind trial in 200 prefrail or frail older adults found that inulin plus oligofructose improved frailty-related measures together with microbiota and metabolite changes [43]. GLP-1 receptor agonists are relevant gut–endocrine comparators rather than fermented-food interventions; a systematic review of Alzheimer disease trials found metabolic signals but no consistent cognitive or amyloid/tau benefit [44]. Collectively, these results are compatible with gut–brain-axis modulation but do not establish clinically meaningful slowing of ageing; future trials need product characterization, long-term follow-up and prespecified functional endpoints.

In summary, LAB-fermented natural compounds have been reported to influence several ageing-relevant processes in experimental models: reducing oxidative and inflammatory damage, recalibrating nutrient sensing in favor of maintenance and repair, and safeguarding mitochondria and macromolecules from dysfunction. These coordinated actions translate into tangible functional benefits in experimental models—from improved endurance and immune function in aged mice [45], to extended lifespan observed in simpler organisms like C. elegans fed with probiotic metabolites [46]. Because most reports are preclinical and use different products and outcome measures, the convergence of these mechanisms should be regarded as a working framework rather than proof of a unified anti-ageing intervention.

## 4. Impacts on Stem Cell Function and Regeneration

Aging not only damages differentiated cells but also impairs adult stem cells, the reservoirs for tissue renewal. A proposed, but incompletely tested, application of LAB-fermented products is support of stem-cell-related tissue maintenance. One way this occurs is indirectly, by creating a more favorable systemic environment (low inflammation, low oxidative stress) for stem cells. Chronic inflammation and ROS can drive stem cell exhaustion and dysfunction [47]. Mitigating these factors could indirectly support stem-cell function, but direct evidence from defined LAB-fermented preparations remains limited. For context, transplantation of microbiota from young mice reduced inflammation and improved haematopoietic stem-cell phenotypes in aged mice [48]; this study supports an inflammation–stem-cell link but does not test a LAB-fermented product.

Beyond this general support, certain fermented botanicals actively stimulate tissue-specific stem cells. A striking case is in the skin and hair. Hair follicles cyclically regenerate from resident stem cells in the bulge region, a process dependent on Wnt/β-catenin signaling [49]. In a murine telogen model, a fermented herbal decoction was associated with altered Wnt/β-catenin-related markers and hair-growth endpoints. In one study [50], a mixture of medicinal herbs was processed by decoction and LAB fermentation, then applied to resting (telogen) phase mouse skin. Mice treated with the fermented extract entered the anagen (growth) phase faster, developing more hair follicles than those treated with non-fermented extrac. Mechanistically, the fermented herbs upregulated Wnt3/10 and β-catenin in the hair follicle niche, which activated bulge stem cells to initiate new hair shaft formation. Markers of follicular stem cell activation and outer root sheath development (Lef1, FGF7) were elevated, consistent with enhanced stem cell differentiation into hair-producing cells. This model suggests a potential association with follicular regeneration, but it cannot establish direct stem-cell activation, an anti-ageing effect, or efficacy in humans.

Another example comes from the nervous system. Neurogenesis in the adult hippocampus (critical for memory) declines with age but can be modulated by diet and gut microbiota. A traditional tonic formula (Sipjeondaebo-tang) fermented with probiotics was associated with hippocampal neurogenesis-related outcomes in a scopolamine-induced amnesia model [51]. Mice receiving the fermented herbal formula showed significantly higher proliferation of neural precursor cells (BrdU-positive cells) in the hippocampus compared to control. Moreover, more newborn neurons survived and matured (evidenced by increased doublecortin and NeuN markers) in the fermented herb group. The treated mice also performed better in memory tests; however, the model does not demonstrate reversal of age-related cognitive decline or a direct causal role for LAB-derived metabolites. The underlying mechanisms included reduced oxidative stress in the brain and upregulation of pro-survival pathways like BDNF/CREB/Akt by the fermented produ. The findings are compatible with, but do not prove, a role for fermentation-associated factors in the gut–brain axis.

LAB themselves or their fermentation products can also directly influence mesenchymal stem cells (MSCs) which regenerate bone, cartilage and other tissues. In an in vitro study, *Lactobacillus delbrueckii* subsp. lactis KUMS-Y33 was reported to suppress adipogenesis and enhance osteogenic markers in human adipose-derived MSCs [52]. In cultured MSCs, the experimental treatment increased expression of RUNX2, collagen I and osteocalcin, and enhanced alkaline phosphatase activity—all markers of osteogenic differentiation. Concurrently, expression of PPARγ and C/EBPα (adipogenic factors) was suppressed, indicating fewer cells becoming fat cells. This in vitro shift in markers does not establish a benefit for skeletal health or osteoporosis in older adults. Similarly, a protein (named P8) secreted by Lactobacillus was found to promote proliferation and migration of MSCs, enhancing wound healing in cell and animal models [53]. These findings provide preliminary mechanistic observations that require confirmation in well-controlled in vivo and clinical studies.

**Figure 3:**
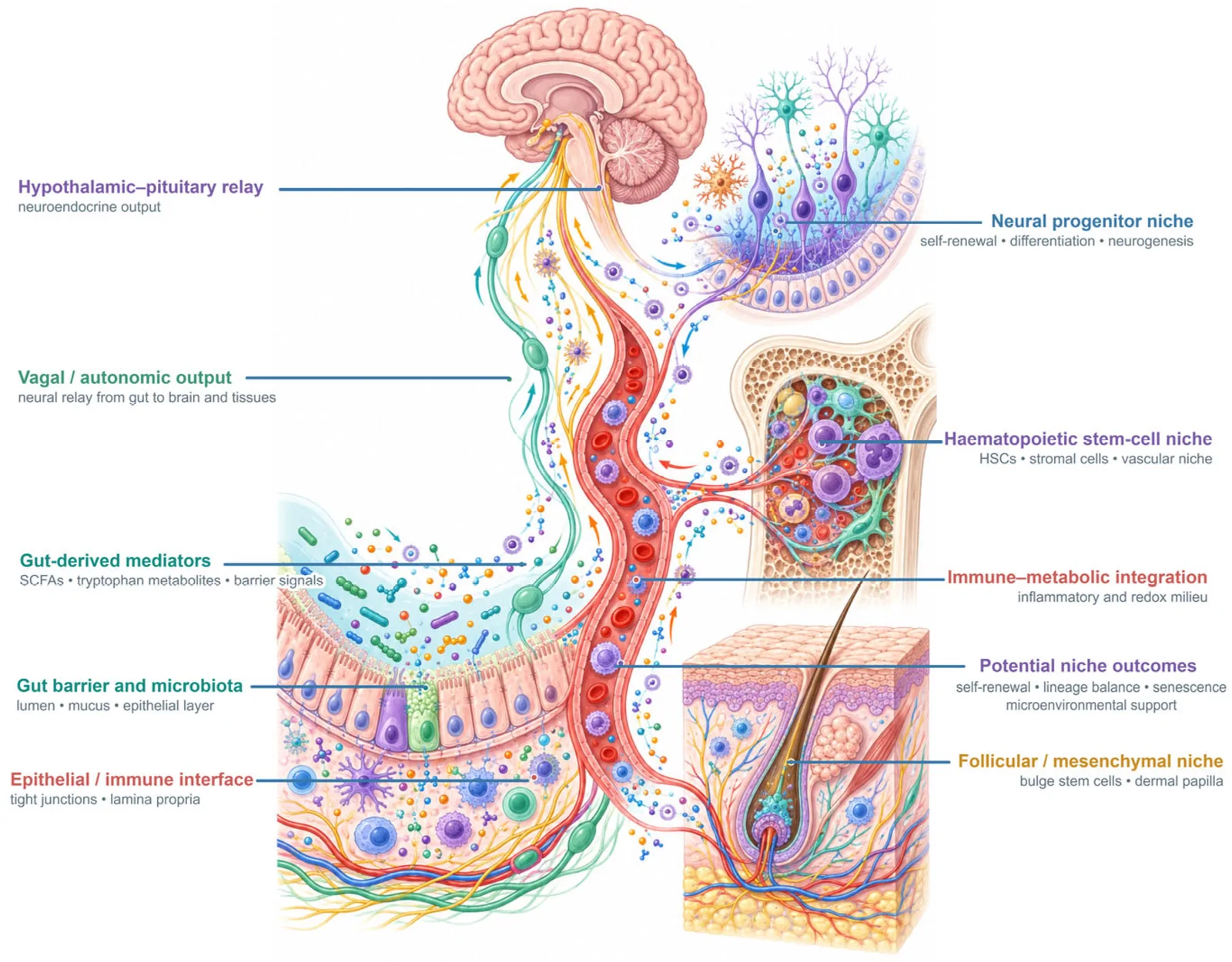
Conceptual mechanism map linking LAB-fermented products, gut–brain-axis communication and stem-cell-related outcomes. Intestinal microbial metabolites (including short-chain fatty acids and tryptophan-derived metabolites) and barrier/immune signals (lower left) may converge on neural, vascular and endocrine relays before reaching neural progenitors (upper right), haematopoietic stem cells in the bone marrow (right centre), and follicular/mesenchymal compartments (lower right). Potential niche outcomes include altered self-renewal, differentiation, senescence and microenvironmental support. Fine trajectories denote interacting hypotheses, not established directional pathways. Evidence is largely mechanistic or preclinical; direct product-specific effects on human stem-cell function require testing.

The gut–brain axis may also shape stem-cell health indirectly by coupling the intestinal ecosystem to neural, endocrine and immune control of regenerative niches (Figure 3). In the gut–bone-marrow axis, short-chain fatty acids, lactate, tryptophan metabolites, microbe-associated molecular patterns and bacterial extracellular vesicles have been proposed as signals that influence systemic cytokine tone and haematopoietic-stem-cell regulation [54]. In aged mice, faecal microbiota transplantation from young donors rejuvenated haematopoietic stem-cell phenotypes while suppressing inflammatory signalling [48]. By analogy, vagal/autonomic and hypothalamic–pituitary–adrenal outputs may modulate neurogenic, haematopoietic and mesenchymal niches, whereas chronic barrier dysfunction and inflammation can favour oxidative stress and stem-cell exhaustion. The direction of effect is context dependent: even a metabolite associated with host benefit may exert different actions after injury or at a given tissue niche. Thus, this network should be viewed as a route for hypothesis testing rather than evidence that any LAB-fermented product directly rejuvenates stem cells.

The current clinical evidence also defines an important gap. The human ageing studies summarized above measured cognitive, inflammatory or frailty-related outcomes rather than in vivo stem-cell number, lineage output or regenerative function [41,42,43]. The most directly relevant clinical setting is haematopoietic-cell transplantation: in a randomized, double-blind phase II trial including allogeneic-transplant recipients and patients receiving induction therapy for acute myeloid leukaemia, oral third-party FMT was safe and improved recovery of microbiota diversity but did not reduce infections [55]. A more recent clinical study showed that FMT engraftment after allogeneic transplantation was donor-dependent; the initial nonrandomized phase supported safety and restoration of microbiota diversity, while the randomized phase remains ongoing [56]. These studies establish microbiota modulation as feasible in a stem-cell-transplant context, not that FMT or LAB-fermented products restore intrinsic stem-cell function. Future trials of standardized LAB-fermented products or other microbiota-directed interventions should incorporate a clinically relevant regenerative outcome and, where feasible, validated surrogate measures of stem-cell or niche status together with microbiome and metabolite profiling.

Taken together, these reports support hypotheses linking selected fermentation products or LAB-derived components to stem-cell-related endpoints. Direct effects, active components, dose–response relationships, and relevance to human tissue maintenance remain insufficiently defined; claims concerning muscle, skin, bone, or neural benefits should therefore remain provisional.

A heterogeneous body of research suggests that LAB fermentation can generate or modify metabolites associated with ageing-relevant and stem-cell-related endpoints. Table 1 summarises representative systems together with their reported effects, evidence level, and principal limitations; it should not be interpreted as evidence of clinical efficacy.

## 5. Cyclic Dipeptides (CDPs) as Bioactive Metabolites

Among the various metabolites produced during LAB fermentation, cyclic dipeptides (CDPs)—also known as 2,5-diketopiperazines—have attracted attention as candidate bioactive compounds, although evidence specific to ageing is limited. CDPs are small ring-shaped molecules consisting of two amino acids cyclized into a dipeptide. LAB can generate CDPs either through dedicated enzymes or as byproducts of protein degradation. CDPs have been detected in some fermented foods; for example, kimchi has been reported to contain LAB-associated CDPs like cyclo(L-Leu-L-Pro) and cyclo(L-Phe-L-Pro) [57]. These compounds are remarkably stable and can withstand harsh conditions (extremes of pH, heat), but stability alone does not establish absorption, exposure, or biological activity in humans.

Sensory and exposure considerations. Several 2,5-diketopiperazines are bitter, metallic, astringent, or mouth-drying food constituents. In roasted cocoa, only a subset of quantified CDPs exceeded their individual bitter taste thresholds, demonstrating that sensory relevance must be assessed by compound-specific concentration-to-threshold comparisons [59]. Comparable quantitative concentration and threshold data are rarely reported for LAB-fermented botanical products; future studies should quantify individual CDPs and relate exposure to both sensory thresholds and biological activity.

Quantitative food-matrix comparators further illustrate why concentration, sensory relevance, and biological activity must be assessed separately. In five commercial beers, seven proline-containing DKPs were detected at concentrations ranging from below the detection limit to approximately 24 ppm, whereas sensory descriptors were assigned in water at 10–50 ppm; the authors questioned whether these DKPs materially contribute to the taste of most beers [60]. In wheat sourdough and bread, cis-cyclo(L-Leu-L-Pro) and cis-cyclo(L-Phe-L-Pro) increased during acidification, but baking temperature was the dominant driver of formation; bread crumb and crust contained approximately 100-fold and 2000-fold, respectively, the pre-baking dough levels. Both were sensory-active in crust but below the minimum inhibitory concentration for antifungal activity in bread [61]. These food-matrix studies do not supply a concentration-to-threshold comparison for LAB-fermented botanical products, but they demonstrate why class-level assumptions about bitterness or antimicrobial function are not justified.

CDPs have a broad spectrum of biological activities that may be relevant to ageing-related processes in experimental systems. Many exhibit strong antioxidant and anti-inflammatory properties, which help curb two major drivers of cellular ageing [62]. For instance, cyclo(His-Pro)—a CDP found in fermented foods and also endogenously in humans—was shown to increase the total antioxidant capacity in neuronal cells and protect them from amyloid-beta toxicity [63]. Other CDPs directly target inflammatory signaling; as mentioned, cyclo(Phe-Pro) (also known as cFP) can suppress NF-κB activation in immune cells [24]. This observation does not establish that cFP decreases chronic inflammatory stress in vivo or in humans. Interestingly, cFP was originally discovered as a quorum-sensing molecule in bacteria, but in mammalian cells it showed a multipotent cytoprotective effect—it not only has anti-tumor and antifungal activities, but also notably reversed radiation-induced damage in animal cell studies. Such observations are hypothesis-generating and should not be extrapolated to anti-ageing efficacy without compound-specific in vivo evidence.

Additionally, some CDPs have been reported to influence cell growth and fate, but direct evidence for stem-cell modulation is lacking. For example, cyclo(Tyr- Phe) exhibiting potent antibacterial activity—especially against *Staphylococcus epidermidis*, surpassing that of chloramphenicol. It also showed strong antitumor effects on A549 lung cancer cells without cytotoxicity to normal fibroblasts, and induced apoptosis via caspase-3 activation [64]. cyclo(Phe-Pro) and cyclo(Leu-Pro) from LAB strongly inhibit pathogenic fungi, bacteria, and even viruses. Antimicrobial activity is not itself evidence of an anti-ageing effect, and its relevance depends on exposure, selectivity, and the host context. For instance, cyclo(L-Phe-L-Pro) from *Lactiplantibacillus plantarum* in kimchi showed potent antiviral activity against influenza viruses, although this antiviral activity has not been shown to protect older individuals [65].

In terms of structure–activity, CDPs are small enough to be bioavailable and some can cross the gut barrier. They may act at local gut level (modulating microbiota or gut immunity) or systemically after absorption. Because individual CDPs can interact with diverse targets, their effects should be assessed compound by compound rather than assumed from the CDP class. However, not all CDPs are beneficial—their effects depend on structure and context. LAB fermentation tends to skew production toward certain CDPs; understanding how fermentation conditions influence the CDP profile is an active area of research [66]. The CDPs highlighted above are candidates for further investigation, not established geroprotective molecules. Dietary exposure to fermented foods or isolated CDPs should not be assumed to confer composite health benefits without evidence for the specific product, dose, and target population. Ongoing studies are isolating individual CDPs from fermented herbs to test their ability to activate stress response pathways like Nrf2 or longevity enzymes like Sirtuins. Future development should first establish CDP identity, exposure, sensory acceptability, safety, and efficacy in relevant human studies.

## 6. Bacterial Extracellular Vesicles (BEVs) as Anti-Ageing Effectors

Beyond small molecules, LAB secrete nanoscale membrane vesicles that carry a cargo of bioactive components. These bacterial extracellular vesicles (BEVs) are typically 20–250 nm lipid bilayer vesicles shed from the bacterial cell surface [66]. For Gram-positive LAB, BEVs encapsulate proteins (including enzymes), peptidoglycan fragments, lipoteichoic acids, metabolites, and even nucleic acids from the bacteria [67]. They can participate in microbe–host communication, although cargo composition and biological effects vary with the producer strain and preparation method. In the context of fermentation, BEVs can be present in fermented products or released by probiotic bacteria in the gut [68].

BEVs can be taken up by mammalian cells in experimental systems, but barrier crossing and tissue distribution must be established for each preparation [69]. BEVs are small enough to be taken up by mammalian cells via endocytosis, allowing their contents to interact with intracellular targets. This uptake can modulate cell behaviour in vitro, but it does not by itself demonstrate targeted delivery in vivo [70]. In a skin ageing study, topical application of *Lactiplantibacillus plantarum* BEVs increased dermal fibroblast density and collagen content, leading to improved skin elasticity and reduced wrinkle depth [58]. The reported skin-related findings are preliminary and should not be interpreted as proof that BEVs reproduce a youthful microbial state or regenerate tissue. Larger controlled studies are required to establish efficacy, dose, product characterisation, and safety.

BEVs have been investigated for immunomodulatory and stress-response effects in cellular and disease-model studies. Certain BEVs carry peptidoglycan and teichoic acid motifs that engage pattern recognition receptors like Toll-like receptor 2 (TLR2) on host cells. Activation of TLR2 by probiotic vesicles can induce an anti-inflammatory state and reinforce barrier functions [71]. High-purity preparations of probiotic BEVs have shown potent anti-inflammatory effects in cell models of gut inflammation [67]. By reducing excessive immune activation, BEVs help maintain tissue homeostasis. Importantly, BEVs can protect cells from oxidative injury. As discussed, *Lactobacillus crispatus*-derived EVs added to stressed placental cells markedly attenuated oxidative stress-induced senescence [37]. These vesicles entered the cells and activated the PI3K/Akt pathway, resulting in increased phosphorylation of Akt and improved mitochondrial function. The net effect was lower rates of cell death and senescence under hydrogen peroxide exposure. Mechanistically, BEV treatment-maintained ATP production and mitochondrial integrity in the challenged cells. Orally administered Lactobacillus-derived EVs ameliorated intestinal inflammation and barrier dysfunction in a mouse colitis model [72]; this disease-model result does not establish systemic distribution or anti-ageing efficacy.

It is worth noting that BEVs encapsulate many of the beneficial factors discussed elsewhere in this review–including enzymes that generate antioxidants, small peptides, and microbial metabolites. Their cargo may contain multiple microbial components, but the active constituent(s) and their reproducibility remain uncertain. However, their nano-scale and particulate nature also raise unique considerations. BEVs could be engineered to target specific cell types or to carry additional therapeutic cargos. On the other hand, safety and dosing of BEVs in humans require careful assessment, as the immune system might react unpredictably to bacterial particles. Available animal studies are insufficient to define a human safety margin. BEVs should therefore be regarded as an emerging bacterial component requiring rigorous characterisation, toxicology, pharmacokinetics, and controlled clinical testing rather than as established postbiotic nanotherapeutics.

## 7. Challenges and Perspectives

The convergence of traditional medicine, microbiology, and gerontology in this field is opening exciting avenues, but several challenges must be addressed to translate these findings into real-world interventions. Natural fermentations can yield variable results depending on strains, substrates, and conditions, so standardization and reproducibility remain central: achieving consistent production of key anti-ageing metabolites (e.g., specific CDPs or ginsenoside conversions) will require stringent process control or biotech routes, and advances in synthetic biology could enable microbial cell factories that reliably produce target compounds at scale. Mechanistic elucidation is equally pressing; although multiple pathways have been implicated (Nrf2 activation, mTOR/AMPK modulation, and others), the field must pinpoint which metabolites drive which effects. Given that fermented products are complex mixtures, isolating active principles—such as a particular CDP or a BEV component—and confirming their roles in vivo is essential for scientific validation. Safety and regulatory hurdles also need careful navigation, as fermented herbal products and postbiotics straddle the boundary between foods and drugs; although some well-characterised strains have food-safety designations, safety cannot be inferred for every strain, botanical matrix, metabolite mixture, or BEV preparation. Dose selection, contaminant control, batch-to-batch metabolite profiling, interaction with medications, and long-term effects in vulnerable populations require rigorous toxicology and clinical assessment. Evidence of efficacy will ultimately hinge on human studies: much of the current support derives from cellular and animal models, so clinical trials are needed to demonstrate tangible outcomes (e.g., improved skin elasticity, physical function, or markers of “biological age”). Because ageing is not a disease, pragmatic designs may focus on age-related conditions—such as sarcopenia, cognitive decline, or skin ageing—as proxies, with early, preregistered, controlled trials using standardised products and prespecified safety and efficacy outcomes laying the groundwork for larger preventive studies. Finally, personalised responses may shape translational success, as interactions between fermented products and a host’s microbiome or genetics can modulate efficacy; tailoring probiotic-fermented interventions to individual microbial profiles or nutritional needs aligns naturally with the broader movement toward personalized nutrition for healthy ageing.

The field is poised to leverage cutting-edge tools to accelerate progress. Metabolomic and transcriptomic profiling can help identify molecular changes associated with fermentation exposure, while genome editing of LAB—such as inserting a cyclodipeptide synthase gene to boost CDP output—offers a route to manipulate yields of selected metabolites, subject to safety and sensory evaluation. Parallel efforts to identify LAB strains with especially potent anti-ageing effects and to unravel their underlying pathways will strengthen the mechanistic foundation upon which well-characterised fermented products can be evaluated in rigorous studies of health-related outcomes.

## 8. Conclusions

Harnessing lactic acid bacteria to ferment natural products offers a useful platform for studying how microbial biotransformation may alter natural-product composition. LAB fermentation can release or transform substrate constituents and generate microbial products; the biological relevance of these changes must be established for each defined preparation. The reviewed studies associate selected preparations with oxidative-stress, inflammatory, nutrient-sensing, mitochondrial, or stem-cell-related endpoints, predominantly in cellular and animal models. These observations justify further research, but do not establish LAB-fermented products as geroprotective interventions. Nevertheless, the translation from bench to bedside is still in early days. Rigorous clinical research is needed to validate efficacy in humans, optimize formulations, and ensure safety over the long term. Priorities are standardised manufacture, chemical and biological characterisation, dose–response studies, long-term safety monitoring, and appropriately powered human trials. Only such studies can determine whether a defined LAB-fermented product provides clinically meaningful benefits beyond established healthy lifestyle measures.

## Figures and Tables

**Table 1:** Representative LAB-fermentation systems, reported effects, evidence levels, and principal limitations.

LAB Strain/Source	Substrate/System	Key Metabolites	Reported Effects/Putative Mechanisms	Evidence Level/Key Limitation	Ref.
*Lactiplantibacillus pentosus*	Lespedeza cuneata	Flavonoids	ROS scavenging; antioxidant activity	In vitro; no human outcome data	[9]
*Lactiplantibacillus plantarum*	Apple pomace	Polyphenols	Redox regulation	Food chemistry/in vitro; no outcome study	[10]
*Lactiplantibacillus plantarum HFY09*	Soymilk	Isoflavones, peptides	Nrf2/KEAP1 activation	D-galactose mouse model; no human outcome data	[17]
*Pediococcus pentosaceus*	Cordyceps militaris	SCFAs	AMPK activation	Mouse model; metabolite attribution unresolved	[20]
Mixed LAB fermentation	Herbal decoction	Polyphenol derivatives	Wnt/β-catenin activation	Mouse skin model; active constituents unresolved	[50]
LAB-fermented herbal formula	Sipjeondaebo-tang	Multi-metabolites	BDNF/CREB/Akt signalling	Mouse amnesia model; no ageing or clinical confirmation	[51]
LAB-derived CDPs	Fermented foods	Cyclo(Phe-Pro), etc.	NF-κB inhibition	Mechanistic/preclinical; source attribution unresolved	[57]
LAB-derived BEVs	Skin system	Extracellular vesicles	Fibroblast activation; ECM synthesis	Skin-cell/cosmetic study; limited human evidence	[58]
LAB-derived BEVs	Cellular model	Extracellular vesicles	PI3K/Akt activation	Cell model; no in vivo ageing confirmation	[37]

Evidence calibration for representative models. In the D-galactose model cited above, mice received *Lactiplantibacillus plantarum* HFY09-fermented soymilk (0.1 mL/10 g body weight) with D-galactose (120 mg/kg body weight); the study measured antioxidant-enzyme activities and oxidative-damage markers in serum, liver, brain, and skin, together with Nrf2-related gene expression [17]. In the separate scopolamine model, fermented Sipjeondaebo-tang was administered orally at 125, 250, or 500 mg/kg for 14 days alongside scopolamine (1 mg/kg), and passive-avoidance/Morris-water-maze performance plus BrdU-, DCX-, and NeuN-related hippocampal readouts were assessed [51]. These exposure-defined outcomes support model-specific biological associations, but they do not establish lifespan extension, identify the causal fermentation-derived component, or demonstrate benefit in human ageing.

## Data Availability

No new data were created or analyzed in this study. Data sharing is not applicable to this article.
